# Mechanical ventilation demographics between 1999 and 2009

**DOI:** 10.1186/cc10720

**Published:** 2012-03-20

**Authors:** DS Sulemanji, E Burns, R Kacmarek

**Affiliations:** 1 Massachusetts General Hospital, Boston, MA, USA

## Introduction

Efforts at many levels are being directed at decreasing the economic burden of mechanical ventilation (MV), its related complications and their consequences. Our aim was to determine the length of MV, reintubation rates and use of noninvasive ventilation (NIV), over a 10-year period.

## Methods

Data were retrospectively collected using the Respiratory Care Services' Database. The number of invasive and noninvasive MV services, their sequence if both were used for a given patient, the duration of the services, and reintubation episodes for years 1999 to 2009 were extracted. Four ICUs were included; surgical, medical, neuro and burn ICUs. If a patient was reintubated within 48 hours of extubation, the case was regarded as a single episode of MV and the duration was calculated accordingly. For NIV, if restarted within 48 hours, it was counted as a single episode as well.

## Results

A total of 19,734 IV and 2,472 NIV episodes were identified during this period. The number of MV episodes increased from 1,660 in 1999 to 2,182 in 2009 with an increasing NIV/IV ratio (from 0.05 to 0.17). In the medical and surgical ICUs, median IV days decreased from 4 to 3 and 3 to 2 days respectively. Overall, 76% of IV episodes lasted <7 days, 14% between 8 and 14 days and 10% >15 days. The number of <7 day IV episodes increased by 8% and >15 day episodes decreased by 7% from 1999 to 2009. The overall reintubation rate was 13.8%. Less than 48-hour reintubation dropped from 14.4% in 1999 to 6.7 in 2009 while more than 48-hour reintubation remained similar (5.6% in 1999 and 6.2% in 2009). NIV use significantly increased over this time - almost quadrupled (from 78 in 1999 to 315 in 2009). The most prominent increase was noted in the surgical and burn ICUs where, in 1999, NIV use was minimal (burn: 0, SICU: 6 and 16 and 131 in 2009). Medical and neuro ICUs doubled their use. A total 52.7% of NIV applications were associated with IV within 48 hours of NIV therapy. The sequence of IV-NIV revealed similar patterns through the years, overall, 58.4% of NIV application followed extubation, 24.2% preceded intubation and 17% was in between.

## Conclusion

We found that the duration of MV decreased, reintubations within 48 hours decreased and the use of NIV increased over this 10-year period. The analysis of outcomes from our data has yet to be completed, but it would not be premature to speculate these results are related to the incorporation of SBT protocols and awaking trials, lesser use of neuromuscular blocking agents as well as extensive application of lung-protective ventilation strategies.

